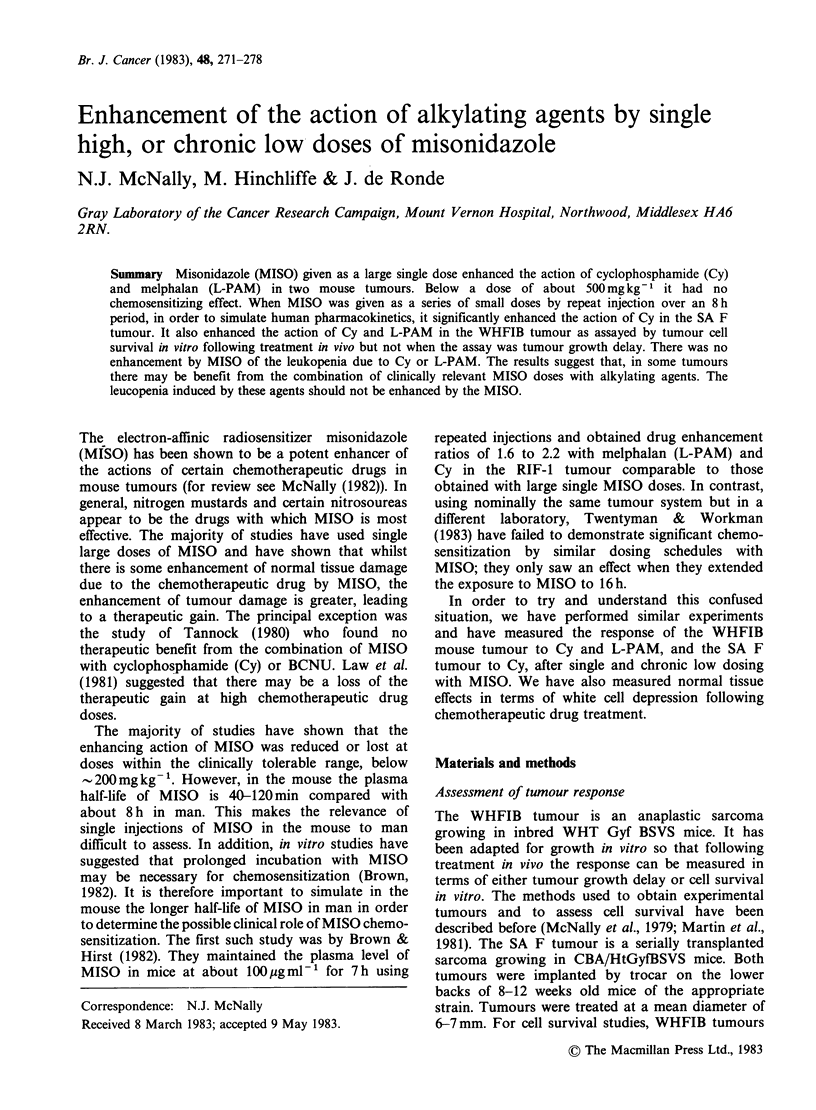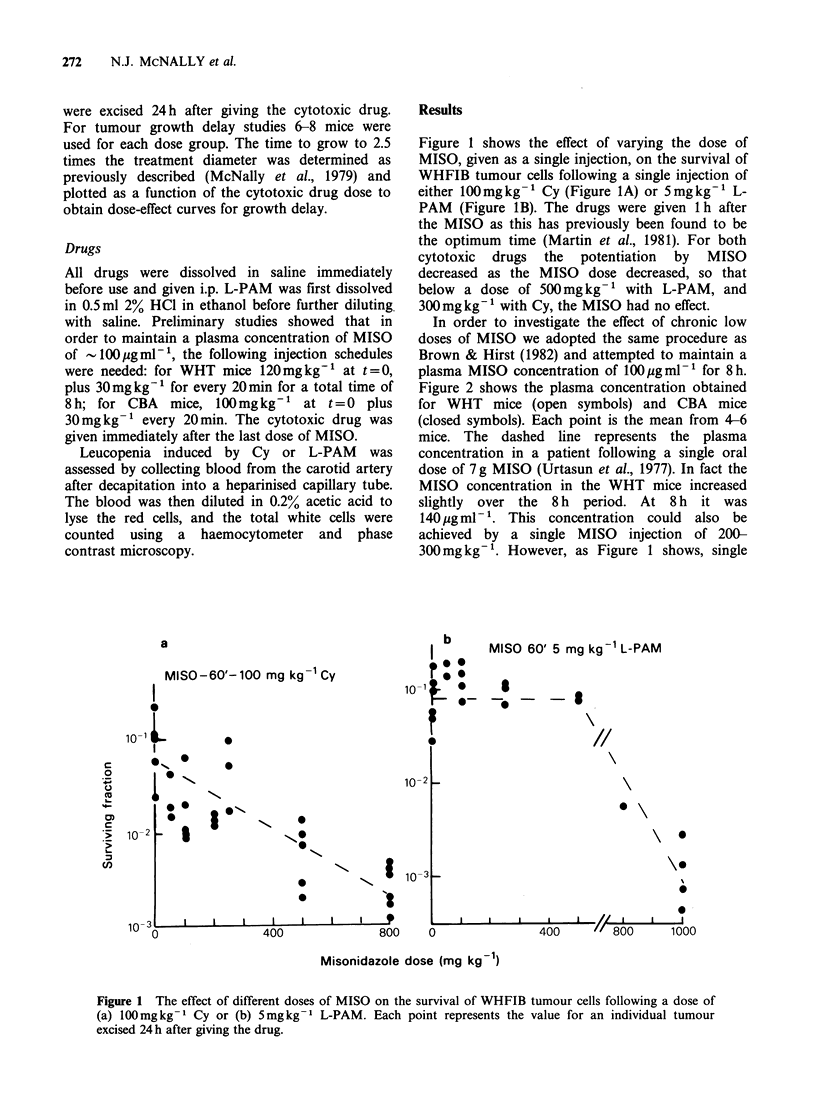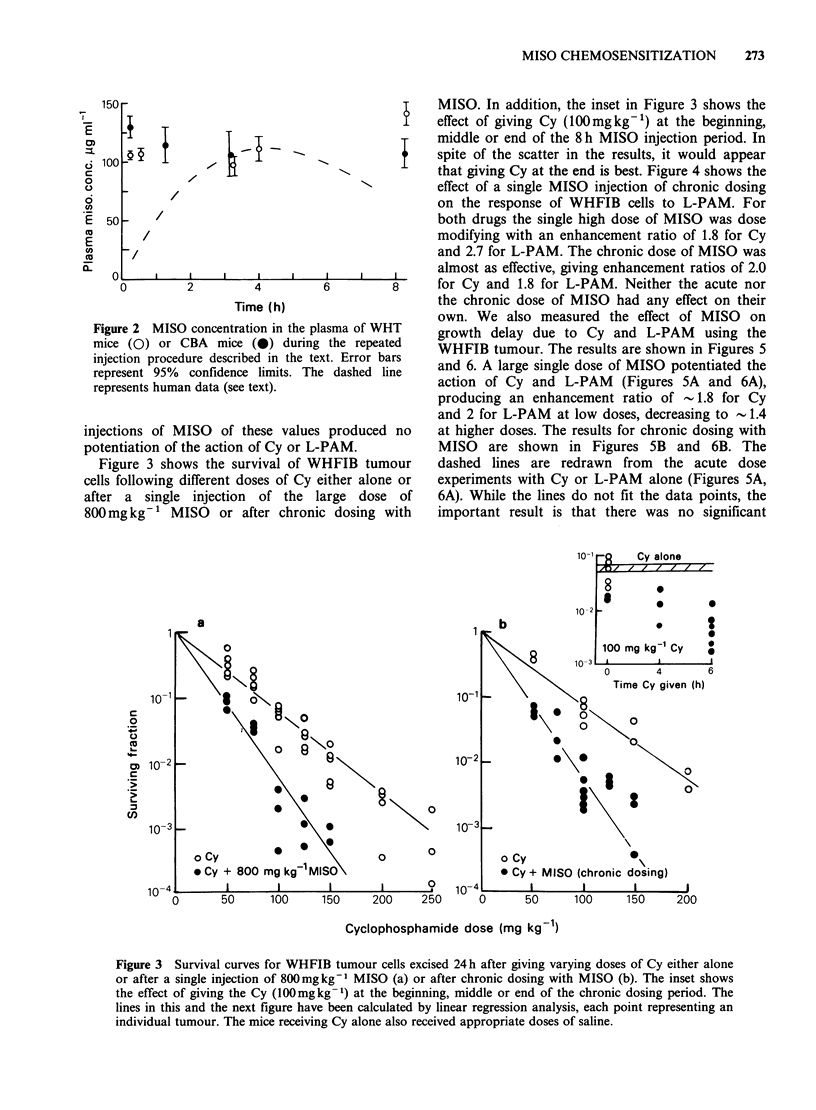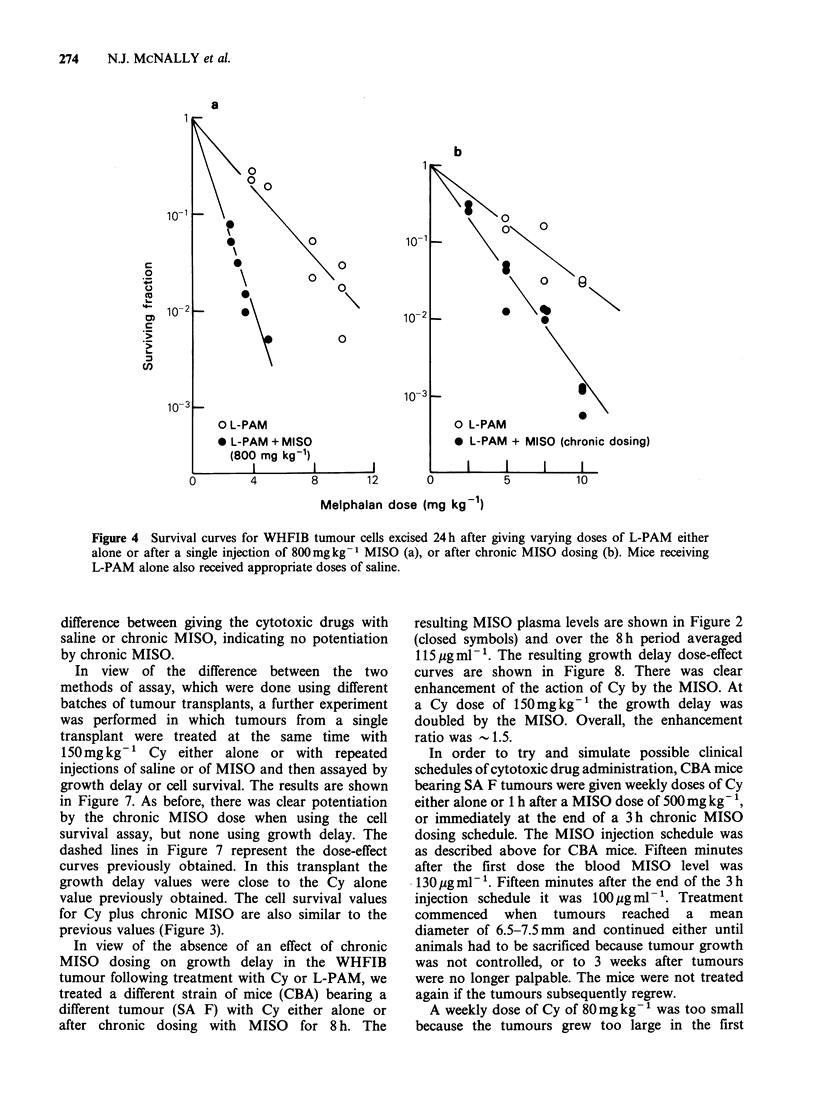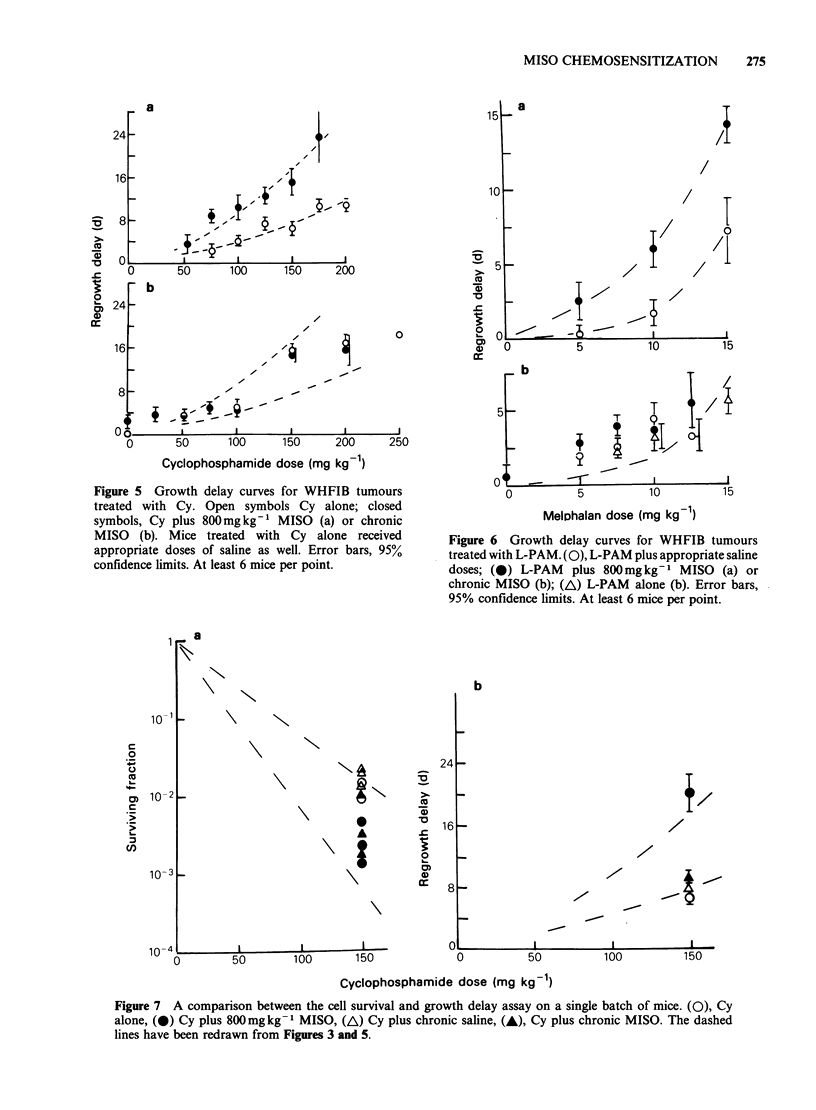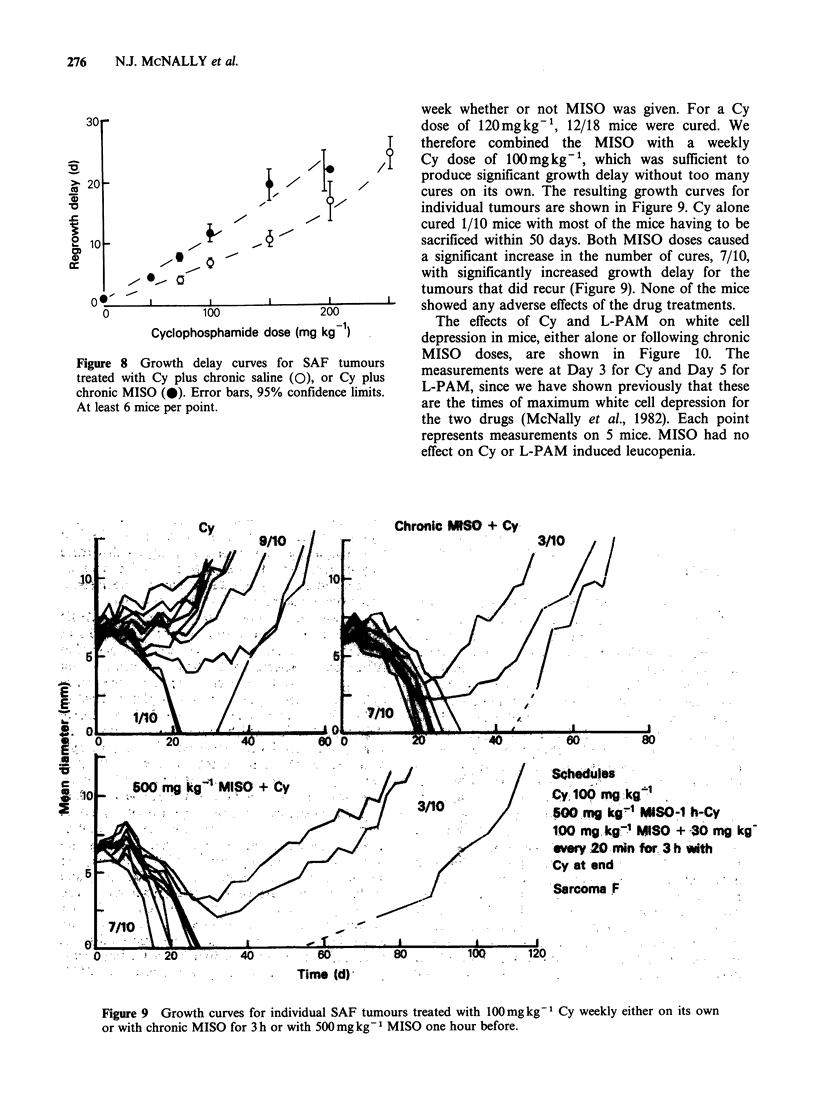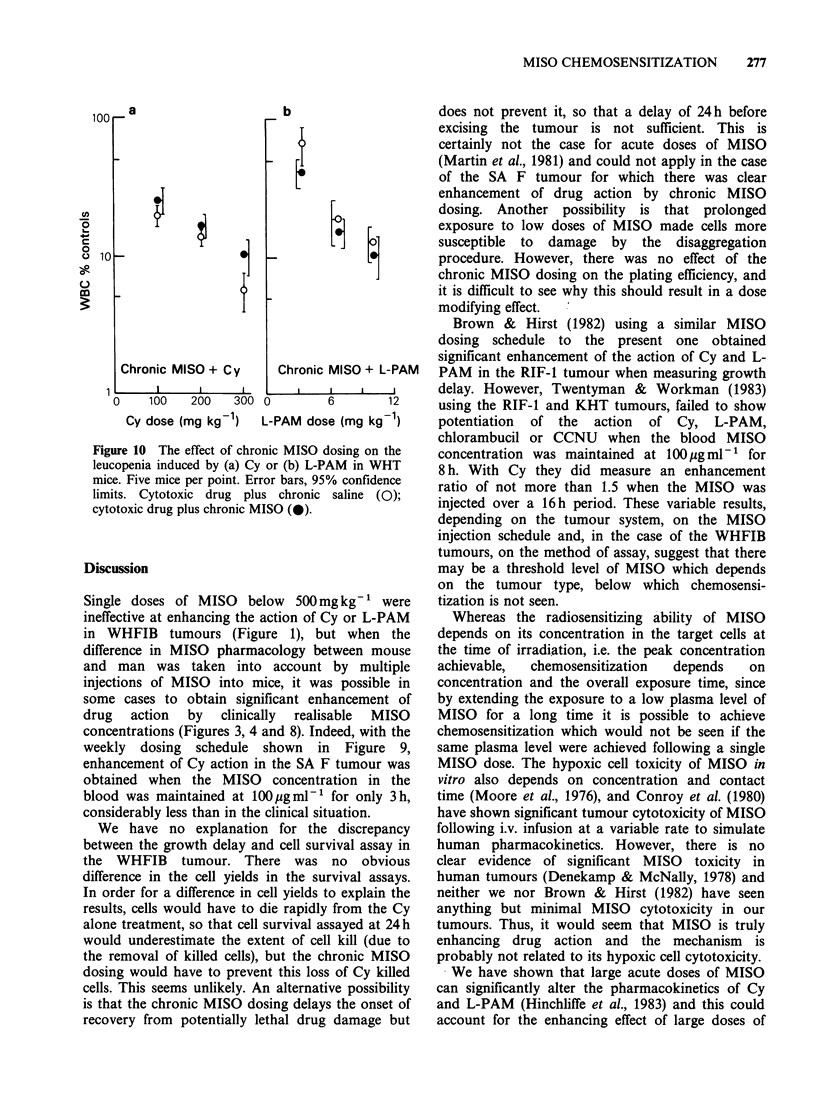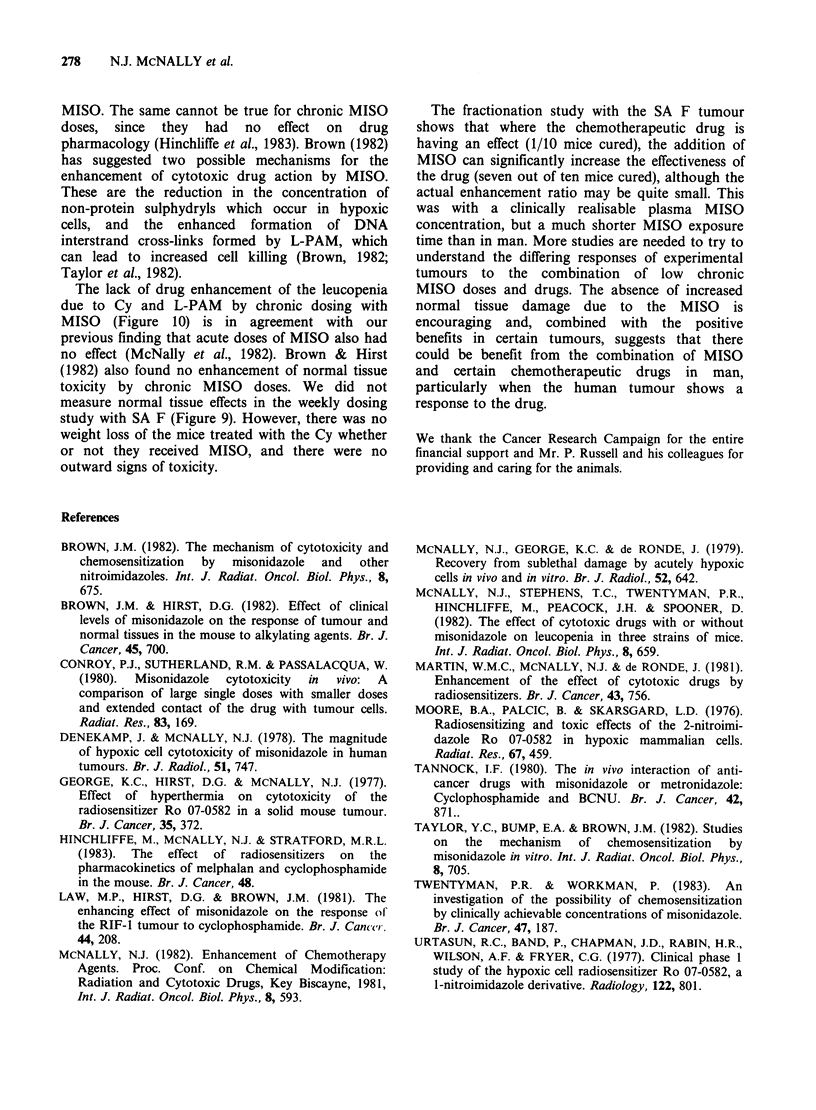# Enhancement of the action of alkylating agents by single high, or chronic low doses of misonidazole.

**DOI:** 10.1038/bjc.1983.182

**Published:** 1983-08

**Authors:** N. J. McNally, M. Hinchliffe, J. de Ronde

## Abstract

Misonidazole (MISO) given as a large single dose enhanced the action of cyclophosphamide (Cy) and melphalan (L-PAM) in two mouse tumours. Below a dose of about 500 mg kg-1 it had no chemosensitizing effect. When MISO was given as a series of small doses by repeat injection over an 8 h period, in order to stimulate human pharmacokinetics, it significantly enhanced the action of Cy in the SA F tumour. It also enhanced the action of Cy and L-PAM in the WHFIB tumour as assayed by tumour cell survival in vitro following treatment in vivo but not when the assay was tumour growth delay. There was no enhancement by MISO of the leukopenia due to Cy or L-PAM. The results suggest that, in some tumours there may be benefit from the combination of clinically relevant MISO doses with alkylating agents. The leucopenia induced by these agents should not be enhanced by the MISO.


*Br. J. Cancer* (1983), **48**, 271–278

### Enhancement of the action of alkylating agents by single high, or chronic low doses of misonidazole

N.J. McNally, M. Hinchliffe & J. de Ronde

*Gray Laboratory of the Cancer Research Campaign, Mount Vernon Hospital, Northwood, Middlesex HA6 2RN.*

**Summary** Misonidazole (MISO) given as a large single dose enhanced the action of cyclophosphamide (Cy) and melphalan (L-PAM) in two mouse tumours. Below a dose of about $500\,mg\,kg^{-1}$ it had no chemosensitizing effect. When MISO was given as a series of small doses by repeat injection over an 8 h period, in order to simulate human pharmacokinetics, it significantly enhanced the action of Cy in the SA F tumour. It also enhanced the action of Cy and L-PAM in the WHFIB tumour as assayed by tumour cell survival *in vitro* following treatment *in vivo* but not when the assay was tumour growth delay. There was no enhancement by MISO of the leukopenia due to Cy or L-PAM. The results suggest that, in some tumours there may be benefit from the combination of clinically relevant MISO doses with alkylating agents. The leucopenia induced by these agents should not be enhanced by the MISO.

The electron-affinic radiosensitizer misonidazole (MISO) has been shown to be a potent enhancer of the actions of certain chemotherapeutic drugs in mouse tumours (for review see McNally (1982)). In general, nitrogen mustards and certain nitrosoureas appear to be the drugs with which MISO is most effective. The majority of studies have used single large doses of MISO and have shown that whilst there is some enhancement of normal tissue damage due to the chemotherapeutic drug by MISO, the enhancement of tumour damage is greater, leading to a therapeutic gain. The principal exception was the study of Tannock (1980) who found no therapeutic benefit from the combination of MISO with cyclophosphamide (Cy) or BCNU. Law *et al.* (1981) suggested that there may be a loss of the therapeutic gain at high chemotherapeutic drug doses.

The majority of studies have shown that the enhancing action of MISO was reduced or lost at doses within the clinically tolerable range, below $\sim 200\,mg\,kg^{-1}$. However, in the mouse the plasma half-life of MISO is 40–120 min compared with about 8 h in man. This makes the relevance of single injections of MISO in the mouse to man difficult to assess. In addition, *in vitro* studies have suggested that prolonged incubation with MISO may be necessary for chemosensitization (Brown, 1982). It is therefore important to simulate in the mouse the longer half-life of MISO in man in order to determine the possible clinical role of MISO chemosensitization. The first such study was by Brown & Hirst (1982). They maintained the plasma level of MISO in mice at about $100\,\mu g\,ml^{-1}$ for 7 h using repeated injections and obtained drug enhancement ratios of 1.6 to 2.2 with melphalan (L-PAM) and Cy in the RIF-1 tumour comparable to those obtained with large single MISO doses. In contrast, using nominally the same tumour system but in a different laboratory, Twentyman & Workman (1983) have failed to demonstrate significant chemosensitization by similar dosing schedules with MISO; they only saw an effect when they extended the exposure to MISO to 16 h.

In order to try and understand this confused situation, we have performed similar experiments and have measured the response of the WHFIB mouse tumour to Cy and L-PAM, and the SA F tumour to Cy, after single and chronic low dosing with MISO. We have also measured normal tissue effects in terms of white cell depression following chemotherapeutic drug treatment.

### Materials and methods

### *Assessment of tumour response*

The WHFIB tumour is an anaplastic sarcoma growing in inbred WHT Gyf BSVS mice. It has been adapted for growth *in vitro* so that following treatment *in vivo* the response can be measured in terms of either tumour growth delay or cell survival *in vitro*. The methods used to obtain experimental tumours and to assess cell survival have been described before (McNally *et al.*, 1979; Martin *et al.*, 1981). The SA F tumour is a serially transplanted sarcoma growing in CBA/HtGyfBSVS mice. Both tumours were implanted by trocar on the lower backs of 8–12 weeks old mice of the appropriate strain. Tumours were treated at a mean diameter of 6–7 mm. For cell survival studies, WHFIB tumours

Correspondence: N.J. McNally
Received 8 March 1983; accepted 9 May 1983.

© The Macmillan Press Ltd., 1983

were excised 24 h after giving the cytotoxic drug. For tumour growth delay studies 6–8 mice were used for each dose group. The time to grow to 2.5 times the treatment diameter was determined as previously described (McNally *et al.*, 1979) and plotted as a function of the cytotoxic drug dose to obtain dose-effect curves for growth delay.

### *Drugs*

All drugs were dissolved in saline immediately before use and given i.p. L-PAM was first dissolved in 0.5 ml 2% HCl in ethanol before further diluting with saline. Preliminary studies showed that in order to maintain a plasma concentration of MISO of $\sim 100\,\mu g\,ml^{-1}$, the following injection schedules were needed: for WHT mice $120\,mg\,kg^{-1}$ at $t=0$, plus $30\,mg\,kg^{-1}$ for every 20 min for a total time of 8 h; for CBA mice, $100\,mg\,kg^{-1}$ at $t=0$ plus $30\,mg\,kg^{-1}$ every 20 min. The cytotoxic drug was given immediately after the last dose of MISO.

Leucopenia induced by Cy or L-PAM was assessed by collecting blood from the carotid artery after decapitation into a heparinised capillary tube. The blood was then diluted in 0.2% acetic acid to lyse the red cells, and the total white cells were counted using a haemocytometer and phase contrast microscopy.

### Results

Figure 1 shows the effect of varying the dose of MISO, given as a single injection, on the survival of WHFIB tumour cells following a single injection of either $100\,mg\,kg^{-1}$ Cy (Figure 1A) or $5\,mg\,kg^{-1}$ L-PAM (Figure 1B). The drugs were given 1 h after the MISO as this has previously been found to be the optimum time (Martin *et al.*, 1981). For both cytotoxic drugs the potentiation by MISO decreased as the MISO dose decreased, so that below a dose of $500\,mg\,kg^{-1}$ with L-PAM, and $300\,mg\,kg^{-1}$ with Cy, the MISO had no effect.

In order to investigate the effect of chronic low doses of MISO we adopted the same procedure as Brown & Hirst (1982) and attempted to maintain a plasma MISO concentration of $100\,\mu g\,ml^{-1}$ for 8 h. Figure 2 shows the plasma concentration obtained for WHT mice (open symbols) and CBA mice (closed symbols). Each point is the mean from 4–6 mice. The dashed line represents the plasma concentration in a patient following a single oral dose of 7 g MISO (Urtasun *et al.*, 1977). In fact the MISO concentration in the WHT mice increased slightly over the 8 h period. At 8 h it was $140\,\mu g\,ml^{-1}$. This concentration could also be achieved by a single MISO injection of 200–$300\,mg\,kg^{-1}$. However, as Figure 1 shows, single

**Figure 1** The effect of different doses of MISO on the survival of WHFIB tumour cells following a dose of (a) $100\,mg\,kg^{-1}$ Cy or (b) $5\,mg\,kg^{-1}$ L-PAM. Each point represents the value for an individual tumour excised 24 h after giving the drug.

**Figure 2** MISO concentration in the plasma of WHT mice (○) or CBA mice (●) during the repeated injection procedure described in the text. Error bars represent 95% confidence limits. The dashed line represents human data (see text).

injections of MISO of these values produced no potentiation of the action of Cy or L-PAM.

Figure 3 shows the survival of WHFIB tumour cells following different doses of Cy either alone or after a single injection of the large dose of $800\,mg\,kg^{-1}$ MISO or after chronic dosing with MISO. In addition, the inset in Figure 3 shows the effect of giving Cy ($100\,mg\,kg^{-1}$) at the beginning, middle or end of the 8 h MISO injection period. In spite of the scatter in the results, it would appear that giving Cy at the end is best. Figure 4 shows the effect of a single MISO injection of chronic dosing on the response of WHFIB cells to L-PAM. For both drugs the single high dose of MISO was dose modifying with an enhancement ratio of 1.8 for Cy and 2.7 for L-PAM. The chronic dose of MISO was almost as effective, giving enhancement ratios of 2.0 for Cy and 1.8 for L-PAM. Neither the acute nor the chronic dose of MISO had any effect on their own. We also measured the effect of MISO on growth delay due to Cy and L-PAM using the WHFIB tumour. The results are shown in Figures 5 and 6. A large single dose of MISO potentiated the action of Cy and L-PAM (Figures 5A and 6A), producing an enhancement ratio of ~1.8 for Cy and 2 for L-PAM at low doses, decreasing to ~1.4 at higher doses. The results for chronic dosing with MISO are shown in Figures 5B and 6B. The dashed lines are redrawn from the acute dose experiments with Cy or L-PAM alone (Figures 5A, 6A). While the lines do not fit the data points, the important result is that there was no significant

**Figure 3** Survival curves for WHFIB tumour cells excised 24 h after giving varying doses of Cy either alone or after a single injection of $800\,mg\,kg^{-1}$ MISO (a) or after chronic dosing with MISO (b). The inset shows the effect of giving the Cy ($100\,mg\,kg^{-1}$) at the beginning, middle or end of the chronic dosing period. The lines in this and the next figure have been calculated by linear regression analysis, each point representing an individual tumour. The mice receiving Cy alone also received appropriate doses of saline.

**Figure 4** Survival curves for WHFIB tumour cells excised 24 h after giving varying doses of L-PAM either alone or after a single injection of $800\,mg\,kg^{-1}$ MISO (a), or after chronic MISO dosing (b). Mice receiving L-PAM alone also received appropriate doses of saline.

difference between giving the cytotoxic drugs with saline or chronic MISO, indicating no potentiation by chronic MISO.

In view of the difference between the two methods of assay, which were done using different batches of tumour transplants, a further experiment was performed in which tumours from a single transplant were treated at the same time with $150\,mg\,kg^{-1}$ Cy either alone or with repeated injections of saline or of MISO and then assayed by growth delay or cell survival. The results are shown in Figure 7. As before, there was clear potentiation by the chronic MISO dose when using the cell survival assay, but none using growth delay. The dashed lines in Figure 7 represent the dose-effect curves previously obtained. In this transplant the growth delay values were close to the Cy alone value previously obtained. The cell survival values for Cy plus chronic MISO are also similar to the previous values (Figure 3).

In view of the absence of an effect of chronic MISO dosing on growth delay in the WHFIB tumour following treatment with Cy or L-PAM, we treated a different strain of mice (CBA) bearing a different tumour (SA F) with Cy either alone or after chronic dosing with MISO for 8 h. The resulting MISO plasma levels are shown in Figure 2 (closed symbols) and over the 8 h period averaged $115\,\mu g\,ml^{-1}$. The resulting growth delay dose-effect curves are shown in Figure 8. There was clear enhancement of the action of Cy by the MISO. At a Cy dose of $150\,mg\,kg^{-1}$ the growth delay was doubled by the MISO. Overall, the enhancement ratio was $\sim 1.5$.

In order to try and simulate possible clinical schedules of cytotoxic drug administration, CBA mice bearing SA F tumours were given weekly doses of Cy either alone or 1 h after a MISO dose of $500\,mg\,kg^{-1}$, or immediately at the end of a 3 h chronic MISO dosing schedule. The MISO injection schedule was as described above for CBA mice. Fifteen minutes after the first dose the blood MISO level was $130\,\mu g\,ml^{-1}$. Fifteen minutes after the end of the 3 h injection schedule it was $100\,\mu g\,ml^{-1}$. Treatment commenced when tumours reached a mean diameter of 6.5–7.5 mm and continued either until animals had to be sacrificed because tumour growth was not controlled, or to 3 weeks after tumours were no longer palpable. The mice were not treated again if the tumours subsequently regrew.

A weekly dose of Cy of $80\,mg\,kg^{-1}$ was too small because the tumours grew too large in the first

**Figure 5** Growth delay curves for WHFIB tumours treated with Cy. Open symbols Cy alone; closed symbols, Cy plus 800 mg kg$^{-1}$ MISO (a) or chronic MISO (b). Mice treated with Cy alone received appropriate doses of saline as well. Error bars, 95% confidence limits. At least 6 mice per point.

**Figure 6** Growth delay curves for WHFIB tumours treated with L-PAM. (○), L-PAM plus appropriate saline doses; (●) L-PAM plus 800 mg kg$^{-1}$ MISO (a) or chronic MISO (b); (△) L-PAM alone (b). Error bars, 95% confidence limits. At least 6 mice per point.

**Figure 7** A comparison between the cell survival and growth delay assay on a single batch of mice. (○), Cy alone, (●) Cy plus 800 mg kg$^{-1}$ MISO, (△) Cy plus chronic saline, (▲), Cy plus chronic MISO. The dashed lines have been redrawn from **Figures 3 and 5**.

**Figure 8** Growth delay curves for SAF tumours treated with Cy plus chronic saline (○), or Cy plus chronic MISO (●). Error bars, 95% confidence limits. At least 6 mice per point.

week whether or not MISO was given. For a Cy dose of $120 \mathrm{mg\,kg^{-1}}$, 12/18 mice were cured. We therefore combined the MISO with a weekly Cy dose of $100 \mathrm{mg\,kg^{-1}}$, which was sufficient to produce significant growth delay without too many cures on its own. The resulting growth curves for individual tumours are shown in Figure 9. Cy alone cured 1/10 mice with most of the mice having to be sacrificed within 50 days. Both MISO doses caused a significant increase in the number of cures, 7/10, with significantly increased growth delay for the tumours that did recur (Figure 9). None of the mice showed any adverse effects of the drug treatments.

The effects of Cy and L-PAM on white cell depression in mice, either alone or following chronic MISO doses, are shown in Figure 10. The measurements were at Day 3 for Cy and Day 5 for L-PAM, since we have shown previously that these are the times of maximum white cell depression for the two drugs (McNally *et al.*, 1982). Each point represents measurements on 5 mice. MISO had no effect on Cy or L-PAM induced leucopenia.

**Figure 9** Growth curves for individual SAF tumours treated with $100 \mathrm{mg\,kg^{-1}}$ Cy weekly either on its own or with chronic MISO for 3 h or with $500 \mathrm{mg\,kg^{-1}}$ MISO one hour before.

**Figure 10** The effect of chronic MISO dosing on the leucopenia induced by (a) Cy or (b) L-PAM in WHT mice. Five mice per point. Error bars, 95% confidence limits. Cytotoxic drug plus chronic saline (○); cytotoxic drug plus chronic MISO (●).

### Discussion

Single doses of MISO below 500 mg kg$^{-1}$ were ineffective at enhancing the action of Cy or L-PAM in WHFIB tumours (Figure 1), but when the difference in MISO pharmacology between mouse and man was taken into account by multiple injections of MISO into mice, it was possible in some cases to obtain significant enhancement of drug action by clinically realisable MISO concentrations (Figures 3, 4 and 8). Indeed, with the weekly dosing schedule shown in Figure 9, enhancement of Cy action in the SA F tumour was obtained when the MISO concentration in the blood was maintained at 100 $\mu$g ml$^{-1}$ for only 3 h, considerably less than in the clinical situation.

We have no explanation for the discrepancy between the growth delay and cell survival assay in the WHFIB tumour. There was no obvious difference in the cell yields in the survival assays. In order for a difference in cell yields to explain the results, cells would have to die rapidly from the Cy alone treatment, so that cell survival assayed at 24 h would underestimate the extent of cell kill (due to the removal of killed cells), but the chronic MISO dosing would have to prevent this loss of Cy killed cells. This seems unlikely. An alternative possibility is that the chronic MISO dosing delays the onset of recovery from potentially lethal drug damage but does not prevent it, so that a delay of 24 h before excising the tumour is not sufficient. This is certainly not the case for acute doses of MISO (Martin *et al.*, 1981) and could not apply in the case of the SA F tumour for which there was clear enhancement of drug action by chronic MISO dosing. Another possibility is that prolonged exposure to low doses of MISO made cells more susceptible to damage by the disaggregation procedure. However, there was no effect of the chronic MISO dosing on the plating efficiency, and it is difficult to see why this should result in a dose modifying effect.

Brown & Hirst (1982) using a similar MISO dosing schedule to the present one obtained significant enhancement of the action of Cy and L-PAM in the RIF-1 tumour when measuring growth delay. However, Twentyman & Workman (1983) using the RIF-1 and KHT tumours, failed to show potentiation of the action of Cy, L-PAM, chlorambucil or CCNU when the blood MISO concentration was maintained at 100 $\mu$g ml$^{-1}$ for 8 h. With Cy they did measure an enhancement ratio of not more than 1.5 when the MISO was injected over a 16 h period. These variable results, depending on the tumour system, on the MISO injection schedule and, in the case of the WHFIB tumours, on the method of assay, suggest that there may be a threshold level of MISO which depends on the tumour type, below which chemosensitization is not seen.

Whereas the radiosensitizing ability of MISO depends on its concentration in the target cells at the time of irradiation, i.e. the peak concentration achievable, chemosensitization depends on concentration and the overall exposure time, since by extending the exposure to a low plasma level of MISO for a long time it is possible to achieve chemosensitization which would not be seen if the same plasma level were achieved following a single MISO dose. The hypoxic cell toxicity of MISO *in vitro* also depends on concentration and contact time (Moore *et al.*, 1976), and Conroy *et al.* (1980) have shown significant tumour cytotoxicity of MISO following i.v. infusion at a variable rate to simulate human pharmacokinetics. However, there is no clear evidence of significant MISO toxicity in human tumours (Denekamp & McNally, 1978) and neither we nor Brown & Hirst (1982) have seen anything but minimal MISO cytotoxicity in our tumours. Thus, it would seem that MISO is truly enhancing drug action and the mechanism is probably not related to its hypoxic cell cytotoxicity.

We have shown that large acute doses of MISO can significantly alter the pharmacokinetics of Cy and L-PAM (Hinchliffe *et al.*, 1983) and this could account for the enhancing effect of large doses of

MISO. The same cannot be true for chronic MISO doses, since they had no effect on drug pharmacology (Hinchliffe *et al.*, 1983). Brown (1982) has suggested two possible mechanisms for the enhancement of cytotoxic drug action by MISO. These are the reduction in the concentration of non-protein sulphydryls which occur in hypoxic cells, and the enhanced formation of DNA interstrand cross-links formed by L-PAM, which can lead to increased cell killing (Brown, 1982; Taylor *et al.*, 1982).

The lack of drug enhancement of the leucopenia due to Cy and L-PAM by chronic dosing with MISO (Figure 10) is in agreement with our previous finding that acute doses of MISO also had no effect (McNally *et al.*, 1982). Brown & Hirst (1982) also found no enhancement of normal tissue toxicity by chronic MISO doses. We did not measure normal tissue effects in the weekly dosing study with SA F (Figure 9). However, there was no weight loss of the mice treated with the Cy whether or not they received MISO, and there were no outward signs of toxicity.

The fractionation study with the SA F tumour shows that where the chemotherapeutic drug is having an effect (1/10 mice cured), the addition of MISO can significantly increase the effectiveness of the drug (seven out of ten mice cured), although the actual enhancement ratio may be quite small. This was with a clinically realisable plasma MISO concentration, but a much shorter MISO exposure time than in man. More studies are needed to try to understand the differing responses of experimental tumours to the combination of low chronic MISO doses and drugs. The absence of increased normal tissue damage due to the MISO is encouraging and, combined with the positive benefits in certain tumours, suggests that there could be benefit from the combination of MISO and certain chemotherapeutic drugs in man, particularly when the human tumour shows a response to the drug.

We thank the Cancer Research Campaign for the entire financial support and Mr. P. Russell and his colleagues for providing and caring for the animals.

### References

BROWN, J.M. (1982). The mechanism of cytotoxicity and chemosensitization by misonidazole and other nitroimidazoles. *Int. J. Radiat. Oncol. Biol. Phys.*, **8**, 675.

BROWN, J.M. & HIRST, D.G. (1982). Effect of clinical levels of misonidazole on the response of tumour and normal tissues in the mouse to alkylating agents. *Br. J. Cancer*, **45**, 700.

CONROY, P.J., SUTHERLAND, R.M. & PASSALACQUA, W. (1980). Misonidazole cytotoxicity *in vivo*: A comparison of large single doses with smaller doses and extended contact of the drug with tumour cells. *Radiat. Res.*, **83**, 169.

DENEKAMP, J. & McNALLY, N.J. (1978). The magnitude of hypoxic cell cytotoxicity of misonidazole in human tumours. *Br. J. Radiol.*, **51**, 747.

GEORGE, K.C., HIRST, D.G. & McNALLY, N.J. (1977). Effect of hyperthermia on cytotoxicity of the radiosensitizer Ro 07-0582 in a solid mouse tumour. *Br. J. Cancer*, **35**, 372.

HINCHLIFFE, M., McNALLY, N.J. & STRATFORD, M.R.L. (1983). The effect of radiosensitizers on the pharmacokinetics of melphalan and cyclophosphamide in the mouse. *Br. J. Cancer*, **48**.

LAW, M.P., HIRST, D.G. & BROWN, J.M. (1981). The enhancing effect of misonidazole on the response of the RIF-1 tumour to cyclophosphamide. *Br. J. Cancer*, **44**, 208.

McNALLY, N.J. (1982). Enhancement of Chemotherapy Agents. Proc. Conf. on Chemical Modification: Radiation and Cytotoxic Drugs, Key Biscayne, 1981, *Int. J. Radiat. Oncol. Biol. Phys.*, **8**, 593.

McNALLY, N.J., GEORGE, K.C. & de RONDE, J. (1979). Recovery from sublethal damage by acutely hypoxic cells *in vivo* and *in vitro*. *Br. J. Radiol.*, **52**, 642.

McNALLY, N.J., STEPHENS, T.C., TWENTYMAN, P.R., HINCHLIFFE, M., PEACOCK, J.H. & SPOONER, D. (1982). The effect of cytotoxic drugs with or without misonidazole on leucopenia in three strains of mice. *Int. J. Radiat. Oncol. Biol. Phys.*, **8**, 659.

MARTIN, W.M.C., McNALLY, N.J. & de RONDE, J. (1981). Enhancement of the effect of cytotoxic drugs by radiosensitizers. *Br. J. Cancer*, **43**, 756.

MOORE, B.A., PALCIC, B. & SKARSGARD, L.D. (1976). Radiosensitizing and toxic effects of the 2-nitroimidazole Ro 07-0582 in hypoxic mammalian cells. *Radiat. Res.*, **67**, 459.

TANNOCK, I.F. (1980). The *in vivo* interaction of anti-cancer drugs with misonidazole or metronidazole: Cyclophosphamide and BCNU. *Br. J. Cancer*, **42**, 871..

TAYLOR, Y.C., BUMP, E.A. & BROWN, J.M. (1982). Studies on the mechanism of chemosensitization by misonidazole *in vitro*. *Int. J. Radiat. Oncol. Biol. Phys.*, **8**, 705.

TWENTYMAN, P.R. & WORKMAN, P. (1983). An investigation of the possibility of chemosensitization by clinically achievable concentrations of misonidazole. *Br. J. Cancer*, **47**, 187.

URTASUN, R.C., BAND, P., CHAPMAN, J.D., RABIN, H.R., WILSON, A.F. & FRYER, C.G. (1977). Clinical phase 1 study of the hypoxic cell radiosensitizer Ro 07-0582, a 1-nitroimidazole derivative. *Radiology*, **122**, 801.